# Stable engraftment of human microbiota into mice with a single oral gavage following antibiotic conditioning

**DOI:** 10.1186/s40168-017-0306-2

**Published:** 2017-08-01

**Authors:** Christopher Staley, Thomas Kaiser, Lalit K. Beura, Matthew J. Hamilton, Alexa R. Weingarden, Aleh Bobr, Johnthomas Kang, David Masopust, Michael J. Sadowsky, Alexander Khoruts

**Affiliations:** 1 BioTechnology Institute, University of Minnesota, St. Paul, MN USA; 2 Center for Immunology, University of Minnesota, 2101 6th St. S.E., Room 3–184, Wallin Medical Biosciences Building, Minneapolis, MN 55414 USA; 3 Department of Microbiology and Immunology, University of Minnesota, Minneapolis, MN USA; 4 Division of Gastroenterology, Department of Medicine, University of Minnesota, Minneapolis, MN USA; 5 Department of Soil, Water, and Climate, University of Minnesota, St. Paul, MN USA

**Keywords:** Antibiotics, Fecal microbiota transplantation, Germ-free, Humanization, Mouse model

## Abstract

**Background:**

Human microbiota-associated (HMA) animal models relying on germ-free recipient mice are being used to study the relationship between intestinal microbiota and human disease. However, transfer of microbiota into germ-free animals also triggers global developmental changes in the recipient intestine, which can mask disease-specific attributes of the donor material. Therefore, a simple model of replacing microbiota into a developmentally mature intestinal environment remains highly desirable.

**Results:**

Here we report on the development of a sequential, three-course antibiotic conditioning regimen that allows sustained engraftment of intestinal microorganisms following a single oral gavage with human donor microbiota. SourceTracker, a Bayesian, OTU-based algorithm, indicated that 59.3 ± 3.0% of the fecal bacterial communities in treated mice were attributable to the donor source. This overall degree of microbiota engraftment was similar in mice conditioned with antibiotics and germ-free mice. Limited surveys of systemic and mucosal immune sites did not show evidence of immune activation following introduction of human microbiota.

**Conclusions:**

The antibiotic treatment protocol described here followed by a single gavage of human microbiota may provide a useful, complimentary HMA model to that established in germ-free facilities. The model has the potential for further in-depth translational investigations of microbiota in a variety of human disease states.

**Electronic supplementary material:**

The online version of this article (doi:10.1186/s40168-017-0306-2) contains supplementary material, which is available to authorized users.

## Background

The human intestinal microbiota is integral to the physiology of its host and plays important roles in immunity and energy metabolism [1, 2]. Antibiotic-induced suppression of gut microbiota can result in loss of microbiota-mediated colonization resistance and allow infections with multidrug-resistant organisms, such a *Clostridium difficile* [3–5]. Furthermore, altered composition and functionality of microbiota has been associated with various disease states, including metabolic syndrome [6, 7], inflammatory bowel disease [8, 9], colorectal cancer [10, 11], and neurodevelopmental disorders [12]. However, establishing causal relationships and performing mechanistic studies on the roles of microbiota in various disease states is often difficult in patient studies.

Animal models can be informative but have limited translational potential since they do not investigate the human microbiota. Therefore, human microbiota-associated (HMA) animal models, primarily using mice, have been increasingly sought to investigate microbe-host relationships in different human disease states [13]. Transfer of human microbiota into germ-free mice by fecal microbiota transplantation (FMT) results in successful transfer of ~85% of genera after 7 days [14], and this HMA mouse model is increasingly being used to study the pathophysiology of human diseases such as obesity [15].

However, there are several fundamental and logistical limitations in using germ-free mice as recipients of human microbiota. The presence of intestinal microbiota is necessary for complete development of the gut and the host immune system, and the absence of microbiota during critical early developmental windows can lead to lasting negative immune and metabolic consequences [16, 17]. Moreover, germ-free facilities require considerable resources to establish and maintain, and germ-free options are not available for many genotypic mouse models [18]. Thus, several groups have tried to overcome these limitations by establishing a HMA animal model following treatment with antibiotics [18–20]. Some attempts suggested that pre-treatment with broad-spectrum antibiotics does not facilitate establishment of exogenous microbiota and may even be deleterious to the input microbial community [20]. Pre-treatment of mice with a 4-day regimen of ciprofloxacin prior to repeated daily gavage with human donor fecal material allowed establishment of only a minor fraction of the human bacterial community [19]. Markedly improved engraftment could be achieved with a more extensive antibiotic regimen that included amphotericin-B, vancomycin, neomycin, metronidazole, and ampicillin [18]. However, this protocol required weekly gavage of human donor material for 12 weeks, and a single gavage was insufficient to establish a HMA model [18].

In this study, we sought to develop an efficient, streamlined antibiotic conditioning regimen that allows substantial and sustained engraftment of human microbiota into specific-pathogen-free (SPF) mice following a single gavage of cryopreserved microbiota. Engraftment of human fecal microbiota was followed by using Illumina-based amplicon sequencing. In addition, the recipient animals were surveyed for signs of systemic and mucosal immune activation and evidence of new cognate antigen encounter by T cells. Engraftment of microbiota in SPF mice was compared with germ-free and restricted flora mice.

## Results

### Transfer of human microbiota to germ-free mice

**Fig. 1:**
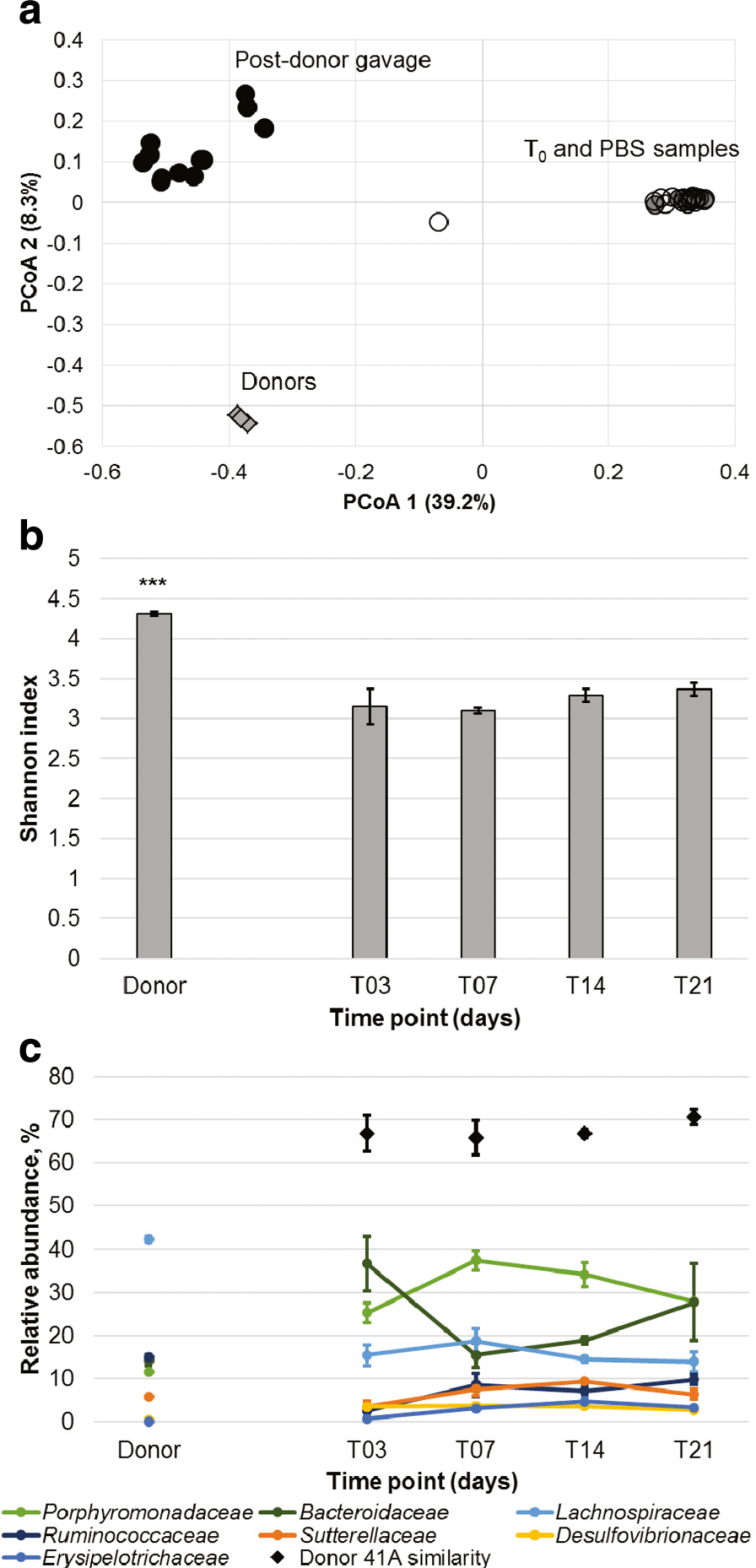
Community composition of germ-free mice gavaged with donor fecal microbiota. **a** Principal coordinate analysis of non-rarefied samples from donor (*diamond*) and mouse samples (*circles*) (*r* ^2^ = 0.78). Legend: *gray*—T_0_ mice, *open*—control mice, *black*—donor-gavaged mice. **b** Shannon indices of bacterial communities in donor and donor-gavaged mice rarefied to 50,456 sequence reads per sample. Shannon indices were averaged among all replicates (*n* = 3) and were significantly higher in donor samples than mice (*P* ≤ 0.001) by Tukey’s post hoc test. **c** Composition of abundant families in donor and donor-gavaged mice communities. Communities were rarefied to 50,456 sequences per sample and abundances of families were averaged among all replicates at each time point (*n* = 3). *Error bars* reflect SEM among replicates. Donor similarity refers to the percent of the community attributable to the donor community, as determined by SourceTracker

The efficacy of transfer of cryopreserved frozen human donor fecal microbiota was initially evaluated in germ-free C57BL/6 mice using a single donor (No. 41) and validated against a control consisting of sterile PBS. Sequencing of germ-free mouse fecal pellets prior to intervention revealed a low number of sequence reads (mean 3028 ± 297) relative to samples post-donor-gavage (167,967 ± 44,543). Due to the low number of sequence reads prior to intervention and following gavage with PBS, non-rarefied sequence data were used to calculate Bray-Curtis dissimilarities for ordination by principal coordinate analysis (PCoA) (Fig. 1a). Control mouse communities showed little divergence from the T_0_ time point, while those from mice gavaged with the donor microbiota showed distinct separation by T_3_ post-gavage, and had a similar position to human donor communities along the *x*-axis.

Bacterial communities were rarefied, by random subsampling, to 50,456 sequence reads per sample for statistical comparisons, thus removing T_0_ and control mice from the dataset. The percent of donor engraftment was determined using the Bayesian SourceTracker algorithm [21], which assigns a percent of the mouse community (sink) to the donor (source) communities based on the presence of common operational taxonomic units (OTUs).

The Shannon indices among human donor communities were significantly greater (4.31 ± 0.03, mean ± SEM) than those of donor-gavaged mice (3.23 ± 0.06; Tukey’s post hoc *P* ≤ 0.001), and Shannon diversity among mouse microbial communities did not differ by time point (Fig. 1b). Evaluation of family-level microbial community composition in colonized mice revealed greater relative abundances of the families *Porphyromonadaceae* and *Bacteroidaceae*, but lower abundances of *Lachnospiraceae* and *Ruminococcaceae* in mouse communities compared to humans (Fig. 1c).

Colonized mouse fecal microbial communities were found to be comprised of approximately 65% of donor taxa (percent of total sequence reads) at T_3,_ and these OTUs expanded to occupy approximately 70% of the community through the 3-week follow-up (Fig. 1c). Only three OTUs were found to be present in all donor and donor-gavaged mouse samples at T_21_, and these were classified as two OTUs within *Bacteroides* and one within the *Subdoligranulum* genus. One OTU within each of *Barnesiella*, *Bacteroides*, and *Subdoligranulum* was shared at T_14_, with the latter two also shared among T_21_ samples. Only one and two OTUs were detected among all donor samples and all T_7_ and T_14_ samples, respectively. Evaluation of community composition by analysis of similarity (ANOSIM), using Bray-Curtis dissimilarities calculated from OTU abundances, revealed that there we no significant differences (at Bonferroni-corrected *α* = 0.005) in beta diversity between donor communities and those of donor-gavaged mice at the individual time points following gavage.

### Minimal transfer of human microbiota to altered Schaedler flora mice

A subsequent experiment was performed using gnotobiotic mice bred with altered Schaedler flora (ASF) [22], a known consortium of eight strains, to determine if colonization by a small number of microorganisms was permissive for the transfer of human microbiota. While mouse communities characterized by next-generation sequencing were more complex than expected, with a mean of 236 ± 22 OTUs among all T_0_ samples, only 7 OTUs were shared. These OTUs were classified as taxa within the *Barnesiella* (2 OTUs), *Clostridium* XIVa, *Lactobacillus*, *Xylanibacter*, *Parabacteroidetes*, and *Sporobacterium*.

**Fig. 2:**
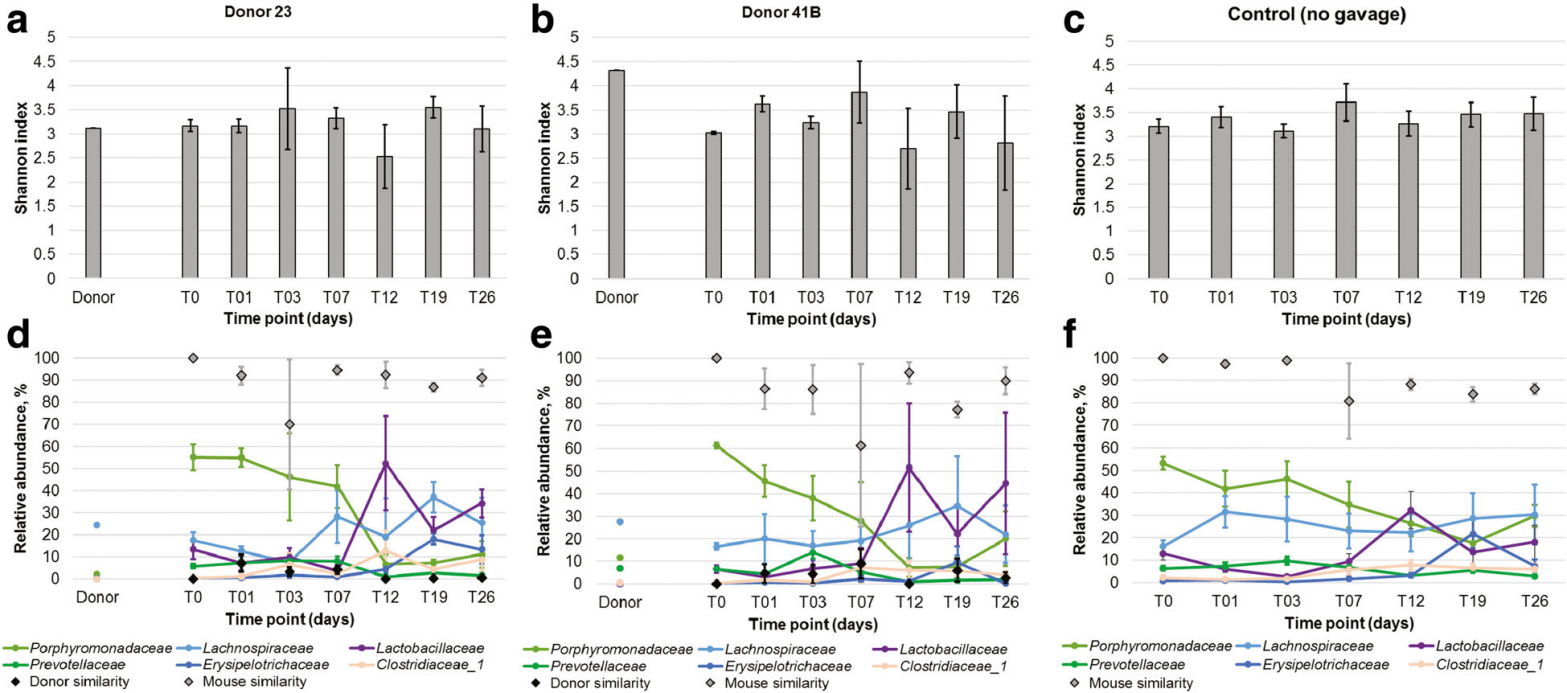
Community composition of ASF mice. **a**–**c** Shannon indices of mice receiving human fecal microbiota from donor 23, donor 41, or no gavage, respectively. Donor samples represented single replicates. Shannon indices are averages from 4, 3, and 5 mice, respectively and *error bars* reflect SEM. **d**–**f** Abundance of predominant families in fecal communities from mice receiving donor 23, 41, or no gavage, respectively. Relative abundances reflect averages among replicates and *error bars* reflect SEM among replicates. Donor and mouse similarity refer to SourceTracker analyses, where the source mouse community was taken as the T_0_ time point. No (0%) donor similarity was detected in the no-gavage control

Two experimental cages of C57BL/6 ASF mice each received gavage with microbiota from a separate human donor (No. 23 or No. 41B, a different lot than that used in experiments with germ-free mice). One additional cage of ASF mice was maintained without gavage. Shannon indices did not vary significantly by ANOVA among donor and mouse samples by time points within a given cage or among cages (Fig. 2a–c; Fisher’s *F* = 0.593). Furthermore, mouse communities were taxonomically distinct from those of their respective donors (Fig. 2d–f).

Ordination of Bray-Curtis dissimilarities among samples revealed some divergence among ASF mice that received donor material from controls, but communities in these mice generally did not appear to become significantly more similar to that of the donors (Additional file 1: Figure S1). ANOSIM analysis, grouping all post-gavage time points by the donor material received, revealed significant differences in community composition between control mouse communities and those of donors (*P* = 0.001). Mice that received donor 23 microbiota maintained communities that were significantly different from that of the donor sample (*P* = 0.002), but these communities did not differ from those of controls or mice that received donor 41B (*P* = 0.044 and 0.230, corrected *α* = 0.005). Conversely, mice that received donor 41 microbiota had a shift in community composition resulting in significant differences from controls (*P* = 0.003), but not the donor community (*P* = 0.012).

SourceTracker found low percentages of similarity (<10%) of microbial communities in post-gavage mice relative to those of either donor 23 or 41B (Fig. 2d–f). T_0_ communities were used to determine the percentage of the community that SourceTracker assigned to the indigenous mouse microbiota, and taking this proportion with that attributed to the donor accounted for approximately 90% of the community in all cages. Using this software, the remainder of the microbial community could not be definitively assigned to a source category.

The primary taxa that were observed to transfer from donors to ASF mice were also detected in ASF mice that did not receive donor gavage (Fig. 2d–f). Among mice receiving donor 23, the predominant families that were attributable to donor at T_3_ and T_7_, when maximum similarity to donor was observed, were the *Bacteroidaceae* (2.58 ± 0.81% of sequence reads) and *Porphyromonadaceae* (0.79 ± 0.27%). For mice that received donor 41, *Bacteroidaceae* (2.21 ± 1.11%), *Clostridiaceae* 1 (1.77 ± 1.94%), and *Porphyromonadaceae* (0.94 ± 0.44%) were the predominant families transferred. No OTU was common among all mouse samples and the donor communities, when evaluations were done at the time of highest observed similarities to donor. However, three of the originally shared OTUs (within *Barnesiella*, *Clostridium* XIVa, and *Lactobacillus*) were still common among 10 of 12 samples at T_26_.

### Limited transfer of human microbiota to SPF mice pre-treated with a single course of antibiotics

Since the gut microbial communities of ASF mice were resistant to the transfer of human fecal microbiota, 1-week-long antibiotic treatments were used as conditioning regimens to disrupt presumed niche specialization causing engraftment resistance. Two different courses of antibiotic treatments were tested, representing antibiotics likely to be systemically absorbed (ampicillin, cefoperazone, and clindamycin) or those not likely to be absorbed (ertapenem, neomycin, and vancomycin). In addition, some mice were treated with a 2-day intestinal purgative to facilitate clearance of residual antibiotics and indigenous microbiota; all mice were gavaged with the same donor material used for germ-free mice (Donor 41A).

All antibiotic treatments significantly reduced Shannon community diversity indices relative to those prior to antibiotic exposure or among donor samples (Tukey’s post hoc *P* < 0.0001). The systemic antibiotic cocktail had variable effects on bacterial community alpha diversity and composition (Additional file 1: Figures S2 and S3), but generally spared members of the *Paenibacillaceae* 1 and *Clostridiaceae* 1 clades. In contrast, the non-absorbable cocktail spared the family *Lactobacillaceae*. For both antibiotic cocktails, the purgative washout also corresponded to an increase in less abundant taxa (Additional file 1: Figure S3), and this effect was considerably more pronounced with the systemic antibiotic cocktail.

Fecal communities in mice that received systemic antibiotics, with or without purgative, following gavage with the same preparation of donor material used for germ-free mice, had similar Shannon indices to donor communities (mean 3.63 to 3.96 for mice and 4.29 ± 0.03 for donor, Tukey’s post hoc *P* > 0.05) by T_21_ and T_14_, respectively. Communities from mice that received systemic antibiotics with purgative had significantly lower Shannon indices than did other mouse groups at T_4_ and T_7_ post-gavage (2.48 ± 0.17 and 3.00 ± 0.21, respectively, compared to means of 3.14 to 3.63 in other groups, *P* < 0.05). In contrast, Shannon diversity did not vary significantly among the other groups examined.

**Fig. 3:**
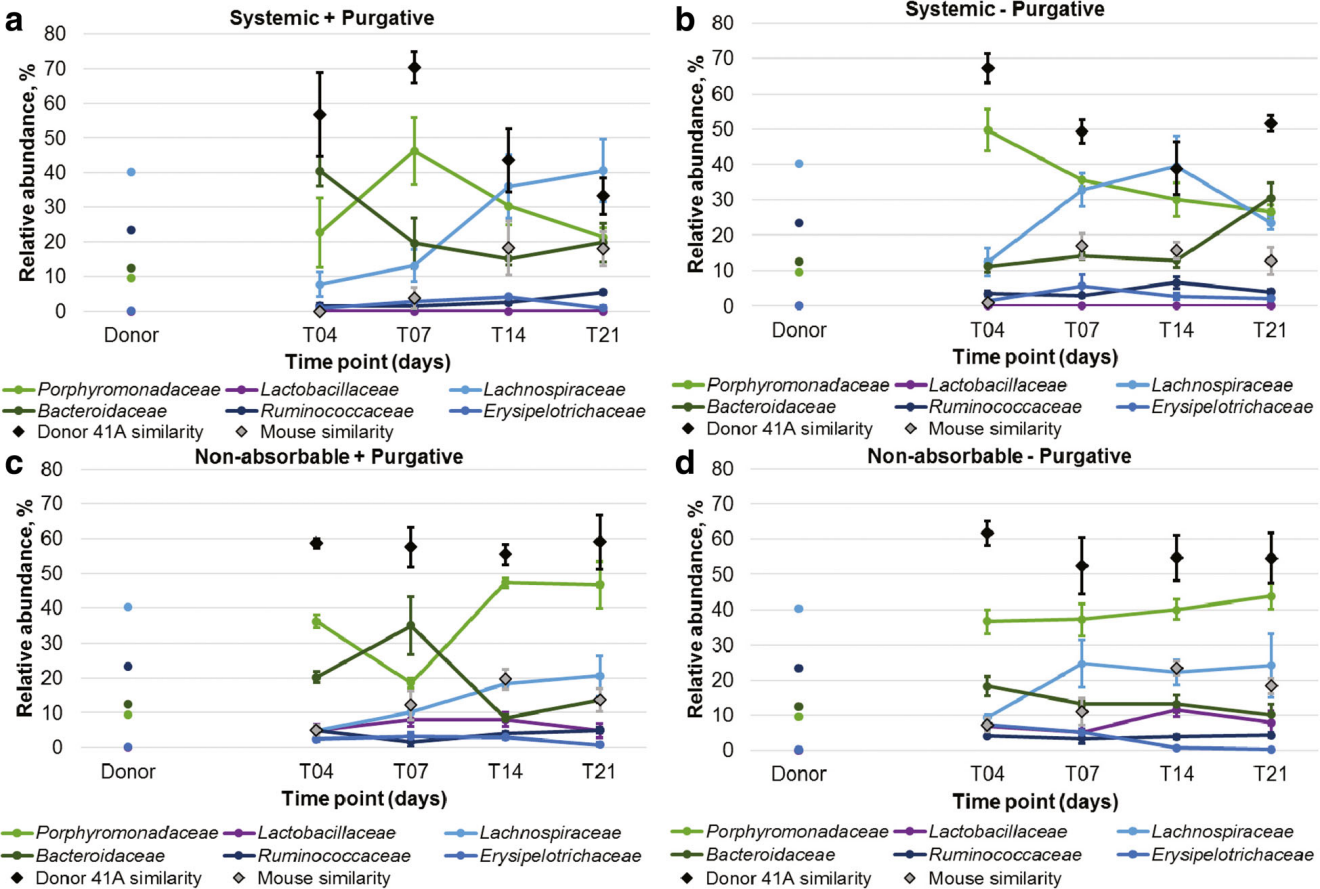
Abundances of predominant families in fecal communities of mice receiving single-course antibiotic treatment. **a** Mice received systemic antibiotics followed by purgative (*n* = 4). **b** Mice received systemic antibiotic without purgative (*n* = 5). **c** Mice received non-absorbable antibiotics followed by purgative (*n* = 4). **d** Mice received non-absorbable antibiotics without purgative (*n* = 4). Relative abundances reflect averages ± SEM among all replicates. Donor and mouse similarity refer to SourceTracker analysis, where the source mouse community was taken as the pre-antibiotic time point. All mice received frozen fecal microbiota from the same donor preparation used in the germ-free mouse experiment

Mice that received systemic antibiotics had communities with significantly greater relative proportions of members of the abundant families *Lachnospiraceae*, *Bacteroidaceae*, *Clostridiaceae* 1, and *Enterobacteriaceae* and lower abundances of *Lactobacillaceae* than did those receiving the non-absorbable cocktail (*P* < 0.05; Table 1, Fig. 3). Similarly, mice that received purgative had greater relative abundances of *Bacteroidaceae*, *Clostridiaceae* 1, and *Enterobacteriaceae*, with lower relative abundances of *Lactobacillaceae* and *Lachnospiraceae* (*P* < 0.05).

**Table 1:** Distribution of abundant families in mouse fecal samples treated with a single course of antibiotics. Family abundances were averaged over all post-gavage time points and are presented as mean ± standard error. ANOVA were performed for each family and, where significant differences were observed, letters denote significant differences by Tukey’s post hoc test (*P* < 0.05)

Antibiotics	Systemic		Non-absorbable		ANOVA
Purgative	Yes	No	Yes	No	*P* value
*Porphyromonadaceae*	30.72 ± 4.24	36 ± 2.76	37.2 ± 3.42	39.19 ± 1.68	0.267
*Lactobacillaceae*	0.03 ± 0.02 A	0.02 ± 0.01 A	6.44 ± 0.86 B	7.62 ± 1.15 B	<0.001
*Lachnospiraceae*	23.29 ± 4.69 AB	27.23 ± 3.45 A	13.46 ± 2.16 B	19.32 ± 2.95 AB	0.027
*Bacteroidaceae*	24.01 ± 3.45 A	16.43 ± 2.02 AB	19.36 ± 3.15 AB	14.02 ± 1.31 B	0.044
*Clostridiaceae_1*	2.80 ± 1.05 A	0.84 ± 0.26 B	0.06 ± 0.04 B	0.05 ± 0.02 B	0.001
*Ruminococcaceae*	2.57 ± 0.49	4.31 ± 0.56	3.86 ± 0.43	3.91 ± 0.36	0.065
*Erysipelotrichaceae*	2.31 ± 0.46	3.05 ± 0.86	2.36 ± 0.41	3.74 ± 0.94	0.464
*Enterobacteriaceae*	5.01 ± 1.30 A	3.08 ± 1.14 AB	4.15 ± 1.31 AB	0.68 ± 0.29 B	0.039
*Acidaminococcaceae*	3.01 ± 0.48	2.25 ± 0.35	3.71 ± 0.67	3.25 ± 0.54	0.197

The percent of donor community similarity among all treatment groups, determined using SourceTracker, was similar to that observed for germ-free mice at T_4_ (mean of 56.7 to 67.2% donor similarity, Tukey’s post hoc test *P* ≥ 0.999; Fig. 3). However, after T_14_ and T_21_ days post-gavage, donor similarity was significantly lower among mice treated with systemic antibiotics (*P* < 0.05) relative to colonized germ-free recipients. Among mice that received the non-absorbable antibiotic cocktail, donor similarity declined slightly, to approximately 55%, but did not differ significantly from the T_4_ time point. Differences in the genera transferred also varied among antibiotic treatment groups and colonized germ-free mice (Table 2). At T_21_, colonized germ-free mice tended to have greater relative abundances of members of the *Bacteroides*, while antibiotic-treated mice harbored greater abundances of *Parabacteroides*, among other genera transferred. However, at T_21_ significantly greater donor similarity was observed by ANOVA in colonized germ-free mice (Fisher’s *F* = 0.026) than in the other mouse groups. No specific OTUs were found to be commonly transferred among all SPF mice. Furthermore, evaluation of the effects of the antibiotic cocktail used or inclusion of purgative revealed that neither parameter significantly influenced the extent of donor microbiota engraftment (*F* = 0.136 and 0.802, respectively).

**Table 2:** Relative abundance (mean ± standard error, %) of predominant genera transferred to mice receiving a single course of antibiotics or germ-free mice. Only genera transferred at ≤1.00% of sequence reads are shown at the T_21_ time point. ANOVA were performed for each family and, where significant differences were observed, letters denote significant differences by Tukey’s post hoc test (*P* < 0.05)

Antibiotic	Systemic		Non-absorbable		Germ-free	ANOVA
Purgative	Yes	No	Yes	No		*P* value
*Barnesiella*	7.89 ± 2.13 B	11.12 ± 2.49 AB	31.80 ± 7.89 A	28.37 ± 2.85 AB	19.03 ± 11.75 AB	0.023
*Parabacteroides*	8.12 ± 0.49 AB	10.70 ± 1.16 A	10.00 ± 0.55 A	11.01 ± 1.52 A	4.34 ± 2.41 B	0.009
*Bacteroides*	13.75 ± 4.10 A	24.00 ± 5.35 A	11.39 ± 1.35 A	8.27 ± 2.62 A	25.37 ± 9.41 A	0.032
*Phascolarctobacterium*	2.17 ± 0.81	1.79 ± 0.54	2.39 ± 1.45	3.00 ± 1.83	0.00 ± 0.00	0.439
*Parasutterella*	0.28 ± 0.19	0.53 ± 0.30	0.89 ± 0.43	1.91 ± 1.37	0.02 ± 0.00	0.316
*Bilophila*	0.33 ± 0.22 B	0.90 ± 0.19 AB	0.85 ± 0.17 AB	0.92 ± 0.42 AB	1.89 ± 0.53 A	0.037
*Alistipes*	0.20 ± 0.16 B	1.90 ± 0.66 A	0.87 ± 0.21 AB	0.52 ± 0.10 AB	1.78 ± 0.68 AB	0.023

### Improved human microbiota engraftment to SPF mice following multiple, sequential antibiotic treatment

In order to more thoroughly disrupt their indigenous gut microbiota, mice were exposed to three courses of antibiotics (7 days each), alternating non-absorbable, systemic, and non-absorbable antibiotic cocktails. The antibiotic treatment was well tolerated and was not associated with any observable negative clinical signs, such as aversion to drinking water or weight loss. Three different donors were used for the experimental groups: frozen fecal preparations from donors No. 36, 41A (the same lot as used in experiments with germ-free mice), and 42 (Additional file 1: Figure S4). In addition, control cages were maintained that included mice receiving either antibiotic treatment alone, without donor gavage, or donor gavage without antibiotic treatment (Additional file 1: Figure S4).

**Fig. 4:**
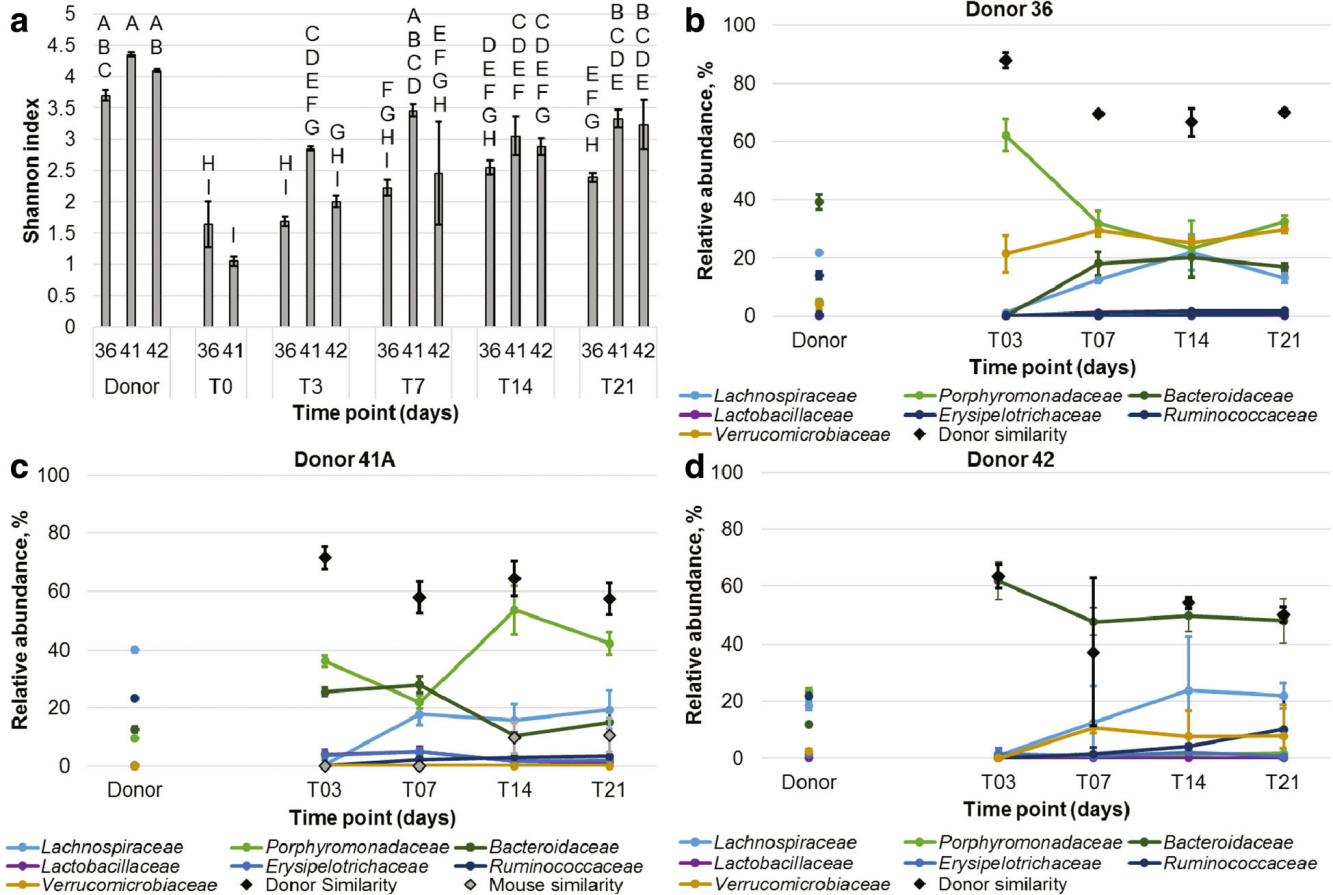
Diversity and abundances of predominant families in fecal communities of multiple-course antibiotic-treated mice. **a** Average Shannon indices of fecal communities from mice receiving antibiotics and donor gavage. *Bars* sharing the same letter did not differ significantly by Tukey’s post hoc test (*P* < 0.05). **b** Predominant families from mice that received gavage from donor 36. **c** Predominant families from mice that received gavage from donor 41. **d** Predominant families from mice that received gavage from donor 42. All values were averaged among replicates from donors (*n* = 3, each), and mice receiving gavage from donors 36, 41, and 42 (*n* = 4, 5, and 2, respectively). Donor and mouse similarity refer to SourceTracker analyses, where the source mouse community was taken as the pre-antibiotic time point for donor 41 only (same lot as used in the germ-free experiment)

Among experimental cages, communities characterized at T_0_, the day of gavage, had significantly lower Shannon indices than did donors or samples from later time points (Tukey’s post hoc *P* ≤ 0.001; Fig. 4a). Shannon indices were significantly greater at T_3_ than at T_0_ (*P* = 0.015). However, by T_7_, differences in alpha diversity were not significantly different from donor communities (*P* ≥ 0.983). Similar to mice treated with a single course of antibiotics, the percentage of donor similarity at T_3_ was high, ranging from 63.4 to 87.9% (mean values; Fig. 4b–d). While these percentages fell slightly over time, high levels of similarity were maintained throughout the 3-week time period, in contrast to the decline in similarity observed with the single-course antibiotic treatment. At T_21_, mice that received the sequential cocktail tended to have a greater percentage of donor engraftment than mice that received single courses of either systemic or non-absorbable antibiotics (*F* = 0.050), although differences in engraftment between mice that received the single-course, non-absorbable cocktail did not differ from those that received the sequential cocktail (Tukey’s *P* = 1.000).

Ordination of Bray-Curtis dissimilarity by PCoA revealed separation of donor and experimental groups from controls and T_0_ samples (Additional file 1: Figure S4). This separation, as evaluated by analysis of molecular variance, was significant (*P* < 0.001), as were differences in community composition, evaluated by ANOSIM (*P* < 0.001). Furthermore, donor communities and those of their corresponding experimental mouse communities did not differ significantly by either test (*P* = 0.002–0.006, Bonferroni-corrected *α* = 0.0009). However, microbial communities in colonized germ-free and antibiotic-treated recipient mice that received fecal material from donor 41A differed significantly by both tests (*P* < 0.001, Additional file 1: Table S1).

Similarity to donor microbiota was comparable for antibiotic-treated and colonized germ-free mice at all time points, with the exception that mice treated with material from donor 36 showed a significantly greater percentage of similarity at T_3_ than did the other groups (*P* ≤ 0.018). In contrast, very low levels of similarity (≤12% of the community) were observed among controls (Additional file 1: Figure S5). SourceTracker analysis identified members of the genus *Bacteroides* to be among the most abundant taxa transferred in germ-free and antibiotic treatment groups (Additional file 1: Figure S6), and *Parabacteroides* (within the family *Porphyromonadaceae*) was also common. Members of the genus *Barnesiella* (within *Porphyromonadaceae*) were common to both groups that received material from donor 41 while members of the genus *Akkermansia* (within *Verrucomicrobiaceae*) were among the most abundant transferred from donor 36.

The resilience of the indigenous SPF mouse community was further investigated and found to recover poorly following antibiotic treatment (Additional file 1: Figure S7). Control mice that received only donor gavage without antibiotics showed much greater similarity to the initial mouse community (89.4–95.0%), through all post-gavage time points. In contrast, by T_21_, only approximately 30% of sequence reads in the control (antibiotics only) community could be attributed to the pre-antibiotic mouse community by SourceTracker analysis, and the majority of these reads (25.52 ± 4.56%) were classified as members of the genus *Anaeroplasma*, with all other genera making only minor (<0.50%) contributions. Furthermore, the rebound of the indigenous community appeared to level off around this final time point. Among the experimental group, a low percentage of the indigenous mouse community appeared to recover (~10%) and did not change from the T_14_ to T_21_ time points. The primary taxa associated with the recovery of the indigenous microbiota were classified to members of the genus *Sporobacterium* (3.28 ± 1.38%) and the group *Clostridium* XIVa (1.14 ± 1.04%) at the T_21_ time point.

### Engraftment of human microbiota into antibiotic-treated SPF mice does not lead to immune activation

Colonization of germ-free mice with commensal microbiota triggers massive activation of the immune system and maturation of the gastrointestinal tract [23–25]. This can be a potential confounding factor in studies that attempt to elucidate differences in functionality between different engrafted microbial communities. However, the immune systems of adult mice housed in the highly hygienic barrier SPF facilities are also relatively immature and resemble that of human newborns [26]. Specifically, adult SPF mice have markedly lower numbers of antigen-experienced T cells with near absence of terminally differentiated effector memory T cells, when compared to adult feral or pet store mice. Therefore, we characterized the T cell phenotype in recipient SPF mice after 1 month following engraftment of human microbiota. The abundance of antigen-experienced and highly differentiated CD8 and CD4 T cells in the spleen (gating strategy shown in Additional file 1: Figure S8), as defined by expression of CD44, CD62L, and killer cell lectin-like receptor subfamily G member 1 (KLRG1), was not altered by antibiotic treatment or engraftment of human microbiota (Additional file 1: Fig. S9A-C). Similarly, there was no effect of antibiotic treatment, with or without engraftment of human microbiota, on the numbers of circulating granulocytes, including neutrophils in peripheral blood (Additional file 1: Fig. S9D and E). In addition, the numbers of hematopoietic (CD45^+^) cells, including CD8α + T cells in the intraepithelial and lamina propria compartments of the small intestine, were also not affected by engraftment of human microbiota (Additional file 1: Fig. S9F and G). Therefore, this limited immunologic survey did not reveal evidence that engrafted human microbiota in SPF mice led to systemic or mucosal immune activation or provided new cognate antigens for effector or memory T cell development.

## Discussion

Although germ-free mice have become adopted as the gold standard animal model in studying the physiologic effects of human gut microbiota associated with different disease states, there remains a need for complimentary models due to a number of limitations intrinsic to the germ-free mice. Previous attempts to achieve substantial engraftment of human microbiota following antibiotic regimens proved difficult and required an intensive schedule of gavage administration with microbiota [18]. Our experience was similar regardless of choice of antibiotics or use of an intestinal purgative. Although donor similarity following conditioning of SPF mice with a cocktail of non-absorbable antibiotics was comparable to colonized germ-free recipients on T_4_, a significant decrease in similarity was observed through the T_21_ time point, especially using the systemic antibiotic cocktail. However, we found that multiple courses of alternating antibiotic cocktails allowed sustained engraftment of human gut microbiota, numerically comparable to that seen with colonized germ-free recipient mice, following a single gavage treatment.

Bacterial communities in germ-free and ASF mice were more complex than expected when characterized by Illumina next-generation sequencing. While no microbial community was expected to be present in germ-free mice, low numbers of sequence reads were obtained, which may reflect sequencing artifacts or transmission of DNA from food pellets. Similarly, while ASF mice were expected to have only eight strains [22], more complex communities were characterized. Low abundances of unexpected taxa in ASF mice were previously reported when characterized using a metatranscriptomic approach [27, 28] and may be due to some degree of contamination in the mouse colonies or misclassification due to short read lengths of DNA used to describe taxonomy from OTUs.

In this study, SourceTracker software was used to determine the extent of donor engraftment using a Bayesian, OTU-based approach [21]. We have previously utilized this approach to measure donor microbiota transfer following FMT to treat recurrent *C. difficile* infection in human patients, [29, 30] and this software has also been used by others to assign invasion scores for transferred taxa [15, 31]. Results of this approach also indicated conservative source assignment following antibiotic treatment, where a considerable percentage of sequence reads were not assigned to the untreated mouse community or the donor prior to or following gavage with donor fecal microbiota. While some degree of uncertainty is not unexpected due to approximately 15% of shared bacterial species between human and mouse hosts [32], the higher unknown percentage following antibiotic treatment may suggest that the OTUs that survive antibiotic treatment are not those that are initially abundant in untreated mouse intestinal communities. Importantly, however, this approach utilizes the most specific taxonomic assignment (OTUs classified at ≥97% similarity) to determine microbiota transfer and may represent a more conservative estimate of transfer than when data are binned to a taxonomic level (e.g., genus).

Antibiotics affect the microbiota of different host species in dissimilar ways; therefore, different cocktails of antibiotics, as well as routes of administration, may prove variably efficacious at reducing and eliminating a host’s indigenous microbiota [33]. We were also concerned that systemically absorbed antibiotics may persist longer in the animals and have deleterious effects on input microbiota. Therefore, we assembled cocktails of antibiotics broadly divided by their systemic bioavailability, as either systemically absorbable or largely non-absorbable. However, while both antibiotic cocktails used in this study proved effective in disrupting the indigenous microbiota to a level that permitted engraftment and early establishment of abundant HMA taxa, residual indigenous species were able to out-compete a considerable proportion of the added human microbiota within the first week following gavage. Antibiotics may also reduce the total microbial density, promoting establishment of human microbiota, but quantitative measures of bacterial density could not be obtained from the data collected. Nevertheless, the potency of the indigenous species in resisting engraftment of human microbiota is supported by the success of the limited ASF consortium in nearly completely resisting establishment of HMA taxa, with several OTUs in the original consortium persisting through T_26_. This is likely due to the fact that similar taxa that are abundantly transferred are already present even in the ASF mice and may further indicate host restrictions to microbiota engraftment associated with differences in host development as a result of a reduced microbial assemblage, similar to that seen with germ-free mice (discussed below) [34, 35].

To overcome this problem, we used a longer conditioning regimen using alternating antibiotic cocktails to achieve more profound elimination of indigenous species. This protocol did result in an environment that was permissive to more sustained establishment of HMA taxa and similar to that in the germ-free mouse model through the time period of this study. We note that using the single-course, non-absorbable cocktail, similar engraftment was observed when compared to mice receiving the extended, alternating cocktail. However, the extended antibiotic course did more thoroughly disrupt similarity to the native mouse community, suggesting this treatment is more likely to result in consistent and extended engraftment independent of the donor preparation. Further testing using multiple donors is necessary to determine the extent to which multiple-course antibiotic treatment is required to establish HMA assemblages versus single-course treatment with the non-absorbable cocktail.

Interestingly, the HMA assemblages in the germ-free and antibiotic-treated mice, which received the same donor preparation, differed significantly. In human FMT experience, donor and recipient strains were shown to coexist following transplantation [36], so it is not surprising that some background indigenous bacteria may alter the total community composition from that observed in germ-free recipients. However, there are also differences between the intestinal environments of germ-free and ASF mice, including the structure of the mucus layer, expression levels of anti-microbial peptides, secreted immunoglobulins, and bile acid composition, [37, 38] and these may also profoundly influence the engraftment potential of different microbial species. In both antibiotic-treated and germ-free models, species within the phylum *Bacteroidetes* were established at greater abundance than were the *Firmicute*s, similar to what was previously reported [18, 19]. This may be due to the capability of some members of the *Bacteroidetes* phylum to preferentially benefit from greater utilization of substrates from the mouse chow diet and endogenous glycoproteins present in intestinal mucus, as well as a greater ability to adhere to the epithelium in the new host [34, 35]. Future studies will be necessary to determine if the antibiotic-treated HMA model functions better, or similar to the germ-free model at recapitulating pathophysiologic states of the human donors.

In our studies we tested only male germ-free mice as recipients, while female mice were used with the antibiotic protocol. It is possible, although unlikely, that the success of microbiota engraftment may vary by recipient sex, and a previous study indicated that genetic background more significantly affected gut microbiota than sexual category [39]. Interestingly, we noted some donor-specific engraftment effects. For example, significantly greater early engraftment of donor taxa was noted in experiments using donor 36 (female). However, long-term differences in donor transfer were not significant, suggesting our protocol is reproducible across different batches of starting donor material.

We found no evidence that transfer of human microbiota into antibiotic-conditioned mice was associated with signs of systemic or mucosal immune activation, at least within the T cell compartments. In contrast, similar immunologic surveys previously demonstrated rapid development of antigen-experienced, highly differentiated T cells following co-housing of SPF and pet store mice [26]. Therefore, it is possible that those changes were caused by actual invasive murine pathogens rather than mere commensals. The human microbiota used in our experiments was prepared from carefully screened and tested healthy human donors. In addition, human microbiota is much less likely to contain pathogens well adapted to the murine host. It is also important to note that at least some microbiota interactions with the host immune system can only be mediated by host-specific microbiota. Thus, colonization of germ-free mice with human microbiota, as opposed to mouse microbiota, cannot fully restore maturation of the intestinal immune system [40, 41], and colonization of germ-free mice with human microbiota does not fully restore microbiota-associated colonization resistance against some pathogens [40]. Lesser ability of human microbes to penetrate the mouse gut barrier was suggested to account for lower T cell proliferation rates in the intestines of germ-free mice colonized with human microbiota relative to those colonized with mouse microbiota [40]. Our results are consistent with this idea. However, in our system, where indigenous mouse microbiota is depleted by an intensive antibiotic regimen in adult animals, the intestinal immune system has already undergone maturation during development. Future experiments will be needed to determine whether certain immune deficiencies arise and persist following intensive conditioning with antibiotics and colonization with human microbiota.

## Conclusions

In summary, here we describe a new antibiotic-based model for establishing human gut microbiota into the intestines of murine hosts, which rivals the germ-free recipient model in extent of engraftment. Clearly, future work is still needed to investigate the longer term and intergenerational durability of engraftment. However, the model already has the potential for many types of experiments and should be easily transferable to different mouse genotypes and investigations of human microbiota associated with different disease states. The platform is economic and broadly available, and we anticipate it will prove complimentary to current models relying exclusively on germ-free mice.

## Methods

### Mice

All mice used were the C57BL/6 genotype and were received at 6–8 weeks of age. Male germ-free mice were bred and maintained in the germ-free facility at the Mayo Clinic (Rochester, MN) [42]. Female altered Schaedler flora (ASF) mice were purchased from Taconic Laboratories (Hudson, NY, USA), housed in autoclaved cages, and were fed irradiated, 18% protein (2918) chow. All other mice were female and were purchased from Charles River Laboratories (Wilmington, MA, USA), maintained in specific-pathogen-free (SPF) cages, and fed 18% protein (2018) chow (non-irradiated version of the 2918 chow). Mice were housed under a 12-h light/dark cycle at 23 °C.

### Antibiotics

All antibiotic solutions were prepared in normal drinking water with each antibiotic at a concentration of 1 mg ml^**−**1^, and all antibiotics remained soluble at this concentration. Antibiotics were provided in 100-ml clear glass sippers (Braintree Scientific, Inc., Braintree, MA, USA). The “systemic antibiotic cocktail” (antibiotics with high degree of systemic absorption) consisted of ampicillin (WG Critical Care, LLC, Paramus, NJ, USA), cefoperazone sodium salt (Sigma-Aldrich Co.), and clindamycin hydrochloride (Fagron, Inc., St. Paul, MN, USA). The “non-absorbable antibiotic cocktail” (antibiotics with relatively small degree of systemic absorption) consisted of Invanz® (ertapenem sodium; Merck and Co., Inc., Whitehouse Station, NJ, USA), neomycin sulfate (Fagron, Inc.), and vancomycin hydrochloride (Mylan Institutional LLC, Rockford, IL, USA). Antibiotic solutions were prepared the day prior to administration to mice in the drinking water and stored at 4 °C.

### Donor material preparation

Donor fecal samples were collected and processed from rigorously screened and tested volunteer standard donors participating in the University of Minnesota donor program for treatment of patients with multiple recurrent *C. difficile* infections, described previously [43]. Fresh fecal samples were collected and processed within 2 h of collection. The material was weighed and homogenized in a sterile commercial blender under N_2_ gas, and particles were removed by passing through stainless steel laboratory sieves with a final pore size of 0.25 mm (WS Tyler, Mentor, OH, USA). The material was centrifuged at 6000×*g* for 15 min, the supernatant was discarded, and the remaining material was resuspended in phosphate buffered saline solution. The concentrated preparation was amended with 10% glycerol (pharmaceutical grade, Sigma-Aldrich Co., St. Louis, MO, USA), and frozen at **−**80 °C until used. Microbial concentrations of preparations were determined microscopically using a Petroff-Hauser counting chamber, and mice received a dose of approximately 10^10^ cells by oral gavage. For all experiments, a total of five preparations were used: two lots from donor 41 collected on different days and one each from donors 23, 36, and 42. An aliquot of fecal sample from each preparation was stored at **−**80 °C for DNA extraction (see below).

### Antibiotic treatment, gavage, and sample collection

Timelines for all mouse experiments are shown in Additional file 1: Fig. S10. Germ-free mice (four mice per cage) received a single oral gavage of 100 μl thawed donor fecal material (donor No. 41; see below) or sterile PBS. Fecal pellets were collected prior to gavage and at days 3, 7, 14, and 21 following gavage and stored at −20 °C until DNA extraction.

Two cages of ASF mice (five mice per cage) received a single oral gavage of 100 μl thawed donor fecal material from one of two donors (donor No. 23 or a different donor No. 41 preparation lot from donor 41 (41B) than that used in germ-free mice; all mice in the same cage received the same material) with one cage maintained without gavage. Mice were gavaged 2 days following receipt. Fecal samples were collected prior to gavage and at 1, 3, 7, 12, 19, and 26 days following gavage.

For the single-course antibiotic experiment, two cages (five mice each) received systemic antibiotics (see below) and two others received non-absorbable antibiotics for 7 days. One cage for each antibiotic treatment received SUPREP bowel prep solution (Braintree Laboratories, Inc., Braintree, MA, USA), comprised of 262 mM sodium sulfate, 38 mM potassium sulfate, and 28 mM magnesium sulfate in drinking water for 2 days following cessation of the antibiotic regimen while the remaining cages received normal drinking water. Mice then received oral gavage of 100 μl of the same thawed donor fecal material given to germ-free mice (donor 41A). Fecal pellets were collected prior to and following antibiotic exposure (prior to bowel prep), following bowel prep (prior to gavage, T_0_), and at days 4, 7, 14, and 21 following gavage.

Mice receiving the three-course antibiotic treatment received non-absorbable antibiotics in drinking water for 7 days, normal drinking water for 2 days, systemic antibiotics in drinking water for 7 days, normal drinking water for 2 days, non-absorbable antibiotics for 7 days, and normal drinking water for 2 days prior to gavage. One cage (five mice) received a single oral gavage of 100 μl of thawed donor fecal material from donor 41A (same used for germ-free), donor 36, or donor 42. Mice in two cages were maintained as no-gavage controls and received the antibiotic regimen without gavage. Mice in two more cages were maintained as no-antibiotic controls and were maintained on normal drinking water for 27 days prior to receiving gavage of 100 μl thawed donor fecal material from either donor 41 or donor 36. Fecal samples were collected prior to antibiotic exposure, following antibiotic exposure (prior to gavage), immediately prior to gavage (T_0_), and at days 3, 7, 14, and 21 following gavage. This experiment was performed in two independent studies to determine reproducibility among donors: the first study included the donor No. 41 treated mice, a no-gavage control cage, and a no-antibiotic control cage; the second study included the remaining donors (Nos. 36 and 42) and control cages. Pre- and post-antibiotic fecal samples were not collected during the second study.

### DNA extraction and sequencing

DNA was extracted from individual mouse fecal pellets (approximately 0.1 g) and from unprocessed donor fecal samples (approximately 0.25 g) using the MoBio (now DNAeasy) PowerSoil DNA Isolation Kit (MoBio Laboratories, Inc., Carlsbad, CA, USA). The V5 + V6 hypervariable regions of the 16S rRNA gene were amplified using the BSF784/R1064 primer set [44] by the University of Minnesota Genomics Center (UMGC, Minneapolis, MN, USA). Illumina (San Diego, CA, USA) sequencing adapters and indices were then added by UMGC using the dual index method [45]. Briefly, amplicons were generated using un-indexed primers. The thermocycling program was 95 °C for 5 min, 25 cycles for 98 °C for 20 s, 55 °C for 15 s, 72 °C for 1 min, and a final extension at 72 °C for 5 min. Indices and flow cell adapters were then added with 10 additional cycles using the same program. Sterile water negative controls were carried through amplification and sequencing. Samples were paired-end sequenced on the Illumina MiSeq (read length of 300 nt) or HiSeq2500 (250 nt) platforms, and results between platforms have been shown to be comparable [46].

### Bioinformatics

All sequence processing was done using mothur software version 1.35.1 [47]. Raw data, as fastq files, were trimmed to 150 nt to remove lower-quality regions and paired-end joined using the fastq-join script [48]. Reads were subsequently quality trimmed to remove those with quality scores <35 over a 50-nt window, homopolymers >8 nt, ambiguous bases, and more than 2-nt mismatches to primer sequences. A low degree of primer mismatch was allowed to account for base changes resulting from the use of a proofing Taq polyermase [45]. High-quality sequences were aligned against the SILVA database (version 119) [49] and subjected to a 2% pre-clustering step to remove likely errors [50]. Chimeric sequences were identified and removed using UCHIME software [51]. Operational taxonomic units (OTUs) were assigned at 97% similarity using the complete-linkage clustering algorithm, and taxonomy was assigned using the version 14 release from the Ribosomal Database Project [52]. Analyses were done using family-level taxonomic assignments, which should be interpreted with some caution due to differences in classification among databases. For example, the family *Porphyromonadaceae* described here may actually represent members of the newly described family S24-7, as delineated in the SILVA database [53]. For comparisons among samples [54], the numbers of sequence reads per sample were rarefied by random subsample to 50,456; 19,156; 12,500; and 20,000 for the germ-free, ASF, single-course antibiotic, and three-course antibiotic experiments, respectively.

The extent of transfer of human-associated bacterial taxa was determined using default parameters of SourceTracker software version 0.9.8 [21]. This software employs an iterative Bayesian approach to determine which OTUs in sink communities are attributable to those in source communities. The fraction of reads that cannot be assigned to a source at a significance threshold of *α* = 0.001 is assigned to an “unknown” category. Communities in donor fecal samples and untreated mouse fecal pellets (where possible) were used as sources.

### Statistics

Alpha diversity of microbial communities was assessed using Shannon indices, calculated using mothur. This index was selected as a common measure of alpha diversity, although it is not reflective of an absolute measure of richness or abundance, and was selected to highlight the relative nature of abundance data presented [55]. Analysis of variance with Tukey’s post hoc test for multiple comparisons was performed using XLSTAT (version 2015.01.0; Addinsoft, Belmont, MA, USA). Bray-Curtis dissimilarity matrices [56] were calculated and used for ordination by principal coordinate analysis [57]. These matrices were also used to assess differences in beta diversity by analysis of similarity (ANOSIM) [58] and significance of sample clustering by analysis of molecular variance (AMOVA) [59]. Bonferroni corrections for multiple comparisons were performed for ANOSIM and AMOVA. All statistics were evaluated at *α* = 0.05, unless corrected for multiple comparisons as noted.

### Immunologic characterization

Mice were euthanized and dissected after 1 month following gavage with human microbiota. Leucocyte isolation from spleen, peripheral blood, and small intestinal intraepithelial and lamina propria compartments were performed as described earlier [60]. Single-cell suspensions were surface-stained with antibodies against CD3 (145-2C11) CD45 (30F-11), CD11b (M1/70), CD11c (N418), MHC II (Ia-Ie) (M5/114.15.2), CD8a (53**–**6.7), CD4 (RM4–5), CD62L (MEL-14), CD44 (IM7), KLRG1 (2F1), CD69 (H1.2F3), and Ly6g (1A8). All the above antibodies were purchased from BD Biosciences (Franklin Lanes, NJ, USA), Biolegend (San Diego, CA, USA), or Affymetrix eBiosciences (Santa Clara, CA, USA). The stained samples were acquired using LSRII or LSR Fortessa flow cytometers (BD) and analyzed with FlowJo software (Treestar, Ashland, OR).

## Additional file

Additional file 1: Supplementary figure and table legends. (DOCX 1197 kb)

## Abbreviations

ASF: Altered Schaedler flora
FMT: Fecal microbiota transplantation
HMA: Human microbiota associated
OTU: Operational taxonomic unit
PCoA: Principal coordinate analysis
SPF: Specific pathogen-free
