# Supplementary material for: Stable engraftment of human microbiota into mice with a single oral gavage following antibiotic conditioning
Source: Microbiome. 2017 Aug 1;5:87. doi: 10.1186/s40168-017-0306-2 (PMC5537947; doi:10.1186/s40168-017-0306-2)
Supplement: Additional file 1: — Supplementary figure and table legends. (DOCX 1197 kb) [file 40168_2017_306_MOESM1_ESM.docx]

**Supplemental Material**

# Stable Engraftment of Human Microbiota into Mice with a Single Oral Gavage Following Antibiotic Conditioning

Christopher Staley^1^, Thomas Kaiser^1^, Lalit K. Beura^2,3^, Matthew J. Hamilton^1^, Alexa R. Weingarden^1^, Aleh Bobr^2,4^ Johnthomas Kang^2,4^, David Masopust^2,3^, Michael J. Sadowsky^1,5,^*, & Alexander Khoruts^1,2,#,^*

^1^BioTechnology Institute, University of Minnesota, St. Paul, Minnesota, USA

^2^Center for Immunology, University of Minnesota, Minneapolis, Minnesota, USA

^3^Department of Microbiology and Immunology, University of Minnesota, Minneapolis, Minnesota, USA

^4^Division of Gastroenterology, Department of Medicine, University of Minnesota, Minneapolis, Minnesota, USA

^5^Department of Soil, Water, and Climate, University of Minnesota, St. Paul, Minnesota, USA

*These authors contributed equally to this work.

^#^Corresponding Author: Alexander Khoruts, 2101 6^th^ St. S.E., Room 3-184, Wallin Medical Biosciences Building, Minneapolis, MN 55414; Telephone: (612)-624-2101, Email: khoru001@umn.edu


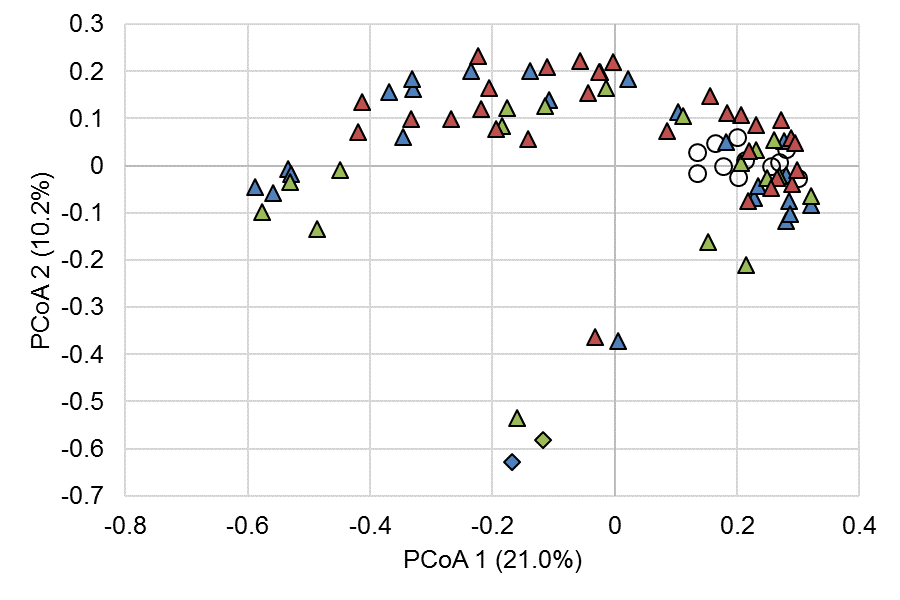


**Figure S1.** Principal coordinate analysis of Bray-Curtis dissimilarity matrices among ASF mice samples and donors (r^2^ = 0.78). Samples collected at T_0_ are shown as open circles. Colors represent the donor, where blue samples are associated with donor 23, green are associated with donor 41, and red are controls. Diamonds represent donor samples and triangles represent post-gavage ASF mice.


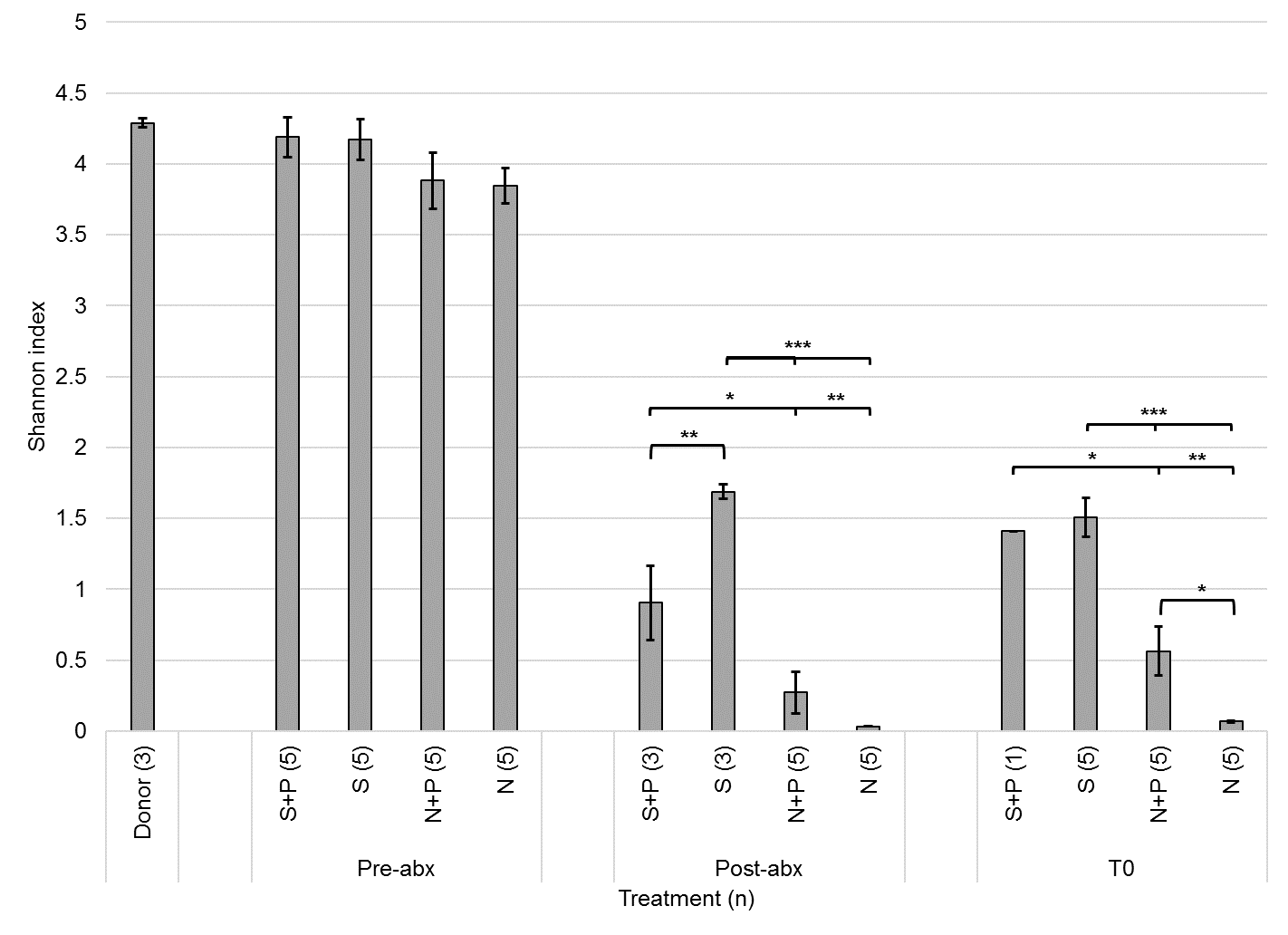


**Figure S2.** Shannon indices for wildtype mouse fecal communities in response to antibiotics. Values are averages of replicates (shown in parentheses) and error bars reflect SEM. T_0_ corresponds to the day of gavage, two days following the post-abx time point. Legend: S – systemic cocktail, N – non-absorbable cocktail, P – purgative.


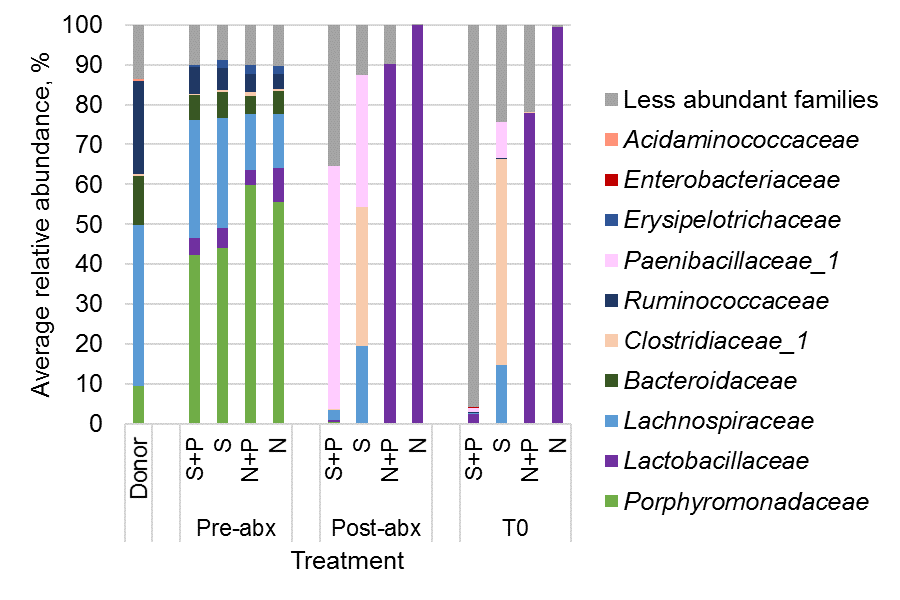


**Figure S3.** Family composition of wildtype mouse fecal communities in response to antibiotics. Values are averages of replicates and error bars reflect SEM. T_0_ corresponds to the day of gavage, two days following the post-abx time point and following purgative administration, if applicable. Legend: S – systemic cocktail, N – non-absorbable cocktail, P – purgative.


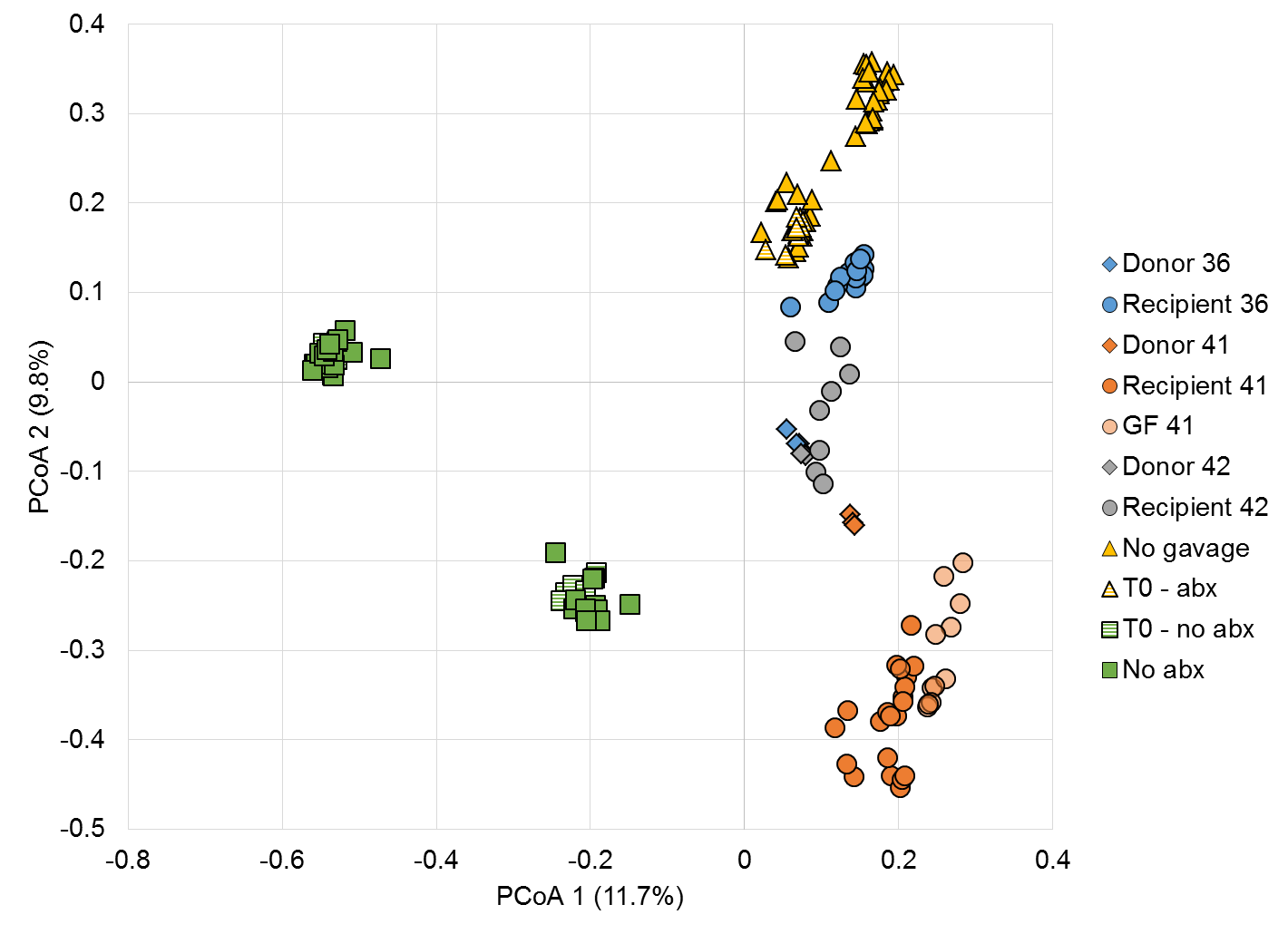


**Figure S4.** Principal coordinate analysis of Bray-Curtis dissimilarities among donor and mouse communities (r^2^ = 0.27). Blue, orange, and gray symbols represent donor samples (♦) and experimental mouse (●) communities. Yellow symbols represent no-gavage controls while green symbols are no-antibiotic controls. The same donor fecal preparation of donor 41 was used for germ-free and antibiotic-exposed mice. GF: germ-free, Recipient: antibiotic-treated recipient of donor gavage, abx: antibiotics.


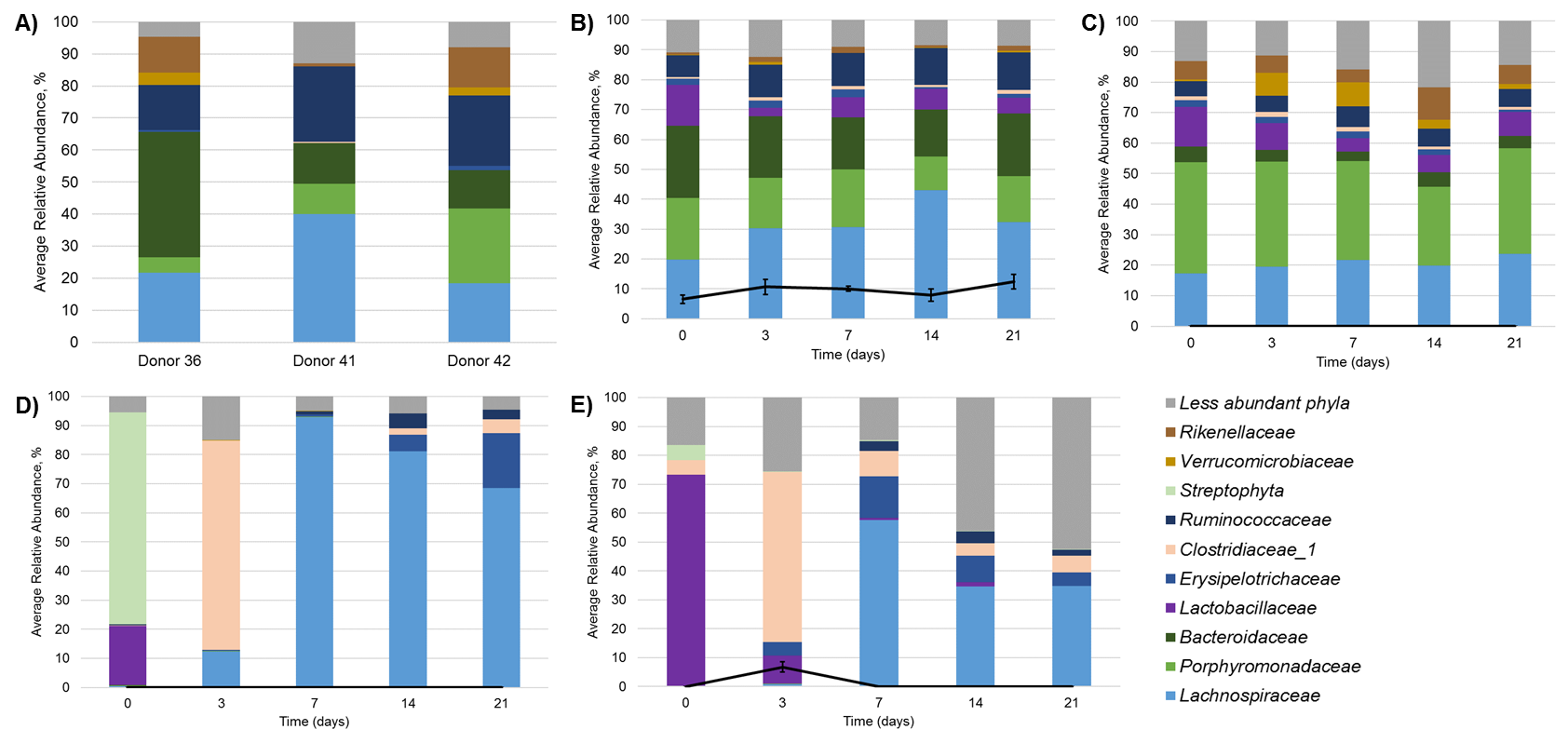


**Figure S5.** Community composition of multi-course antibiotic controls. A) Donor communities (n=3 each), B) mice received gavage of fecal microbiota from donor 36 without prior antibiotic treatment (n=3), C) mice received gavage of fecal microbiota from donor 41 without prior antibiotic treatment (n=5), D & E) mice received antibiotics but no donor gavage (n=5 each; experiments conducted independently). Black lines reflect percent of donor similarity, determined by SourceTracker, and error bars reflect SEM.


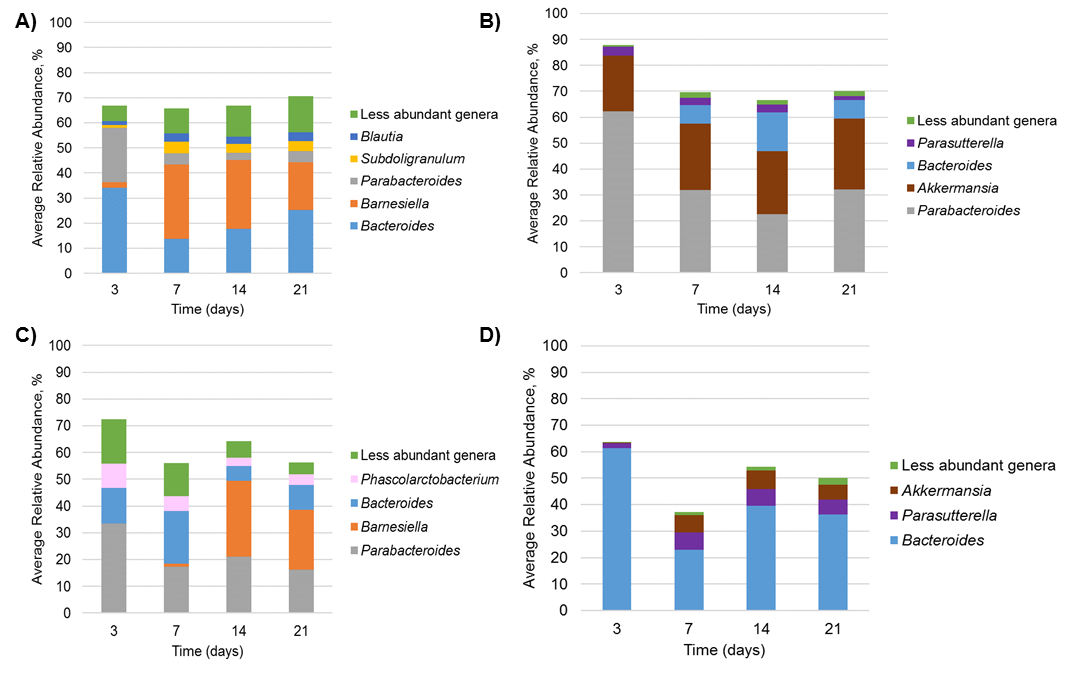


**Figure S6.** Genera associated with donor source contributions. A) Germ-free mice that received donor 41, B) antibiotic-treated mice that received donor 36, C) antibiotic-treated mice that received donor 41, D) antibiotic-treated mice that received donor 42. Genera were identified by SourceTracker and averaged among all replicates (n=3, 4, 5, and 2, respectively).


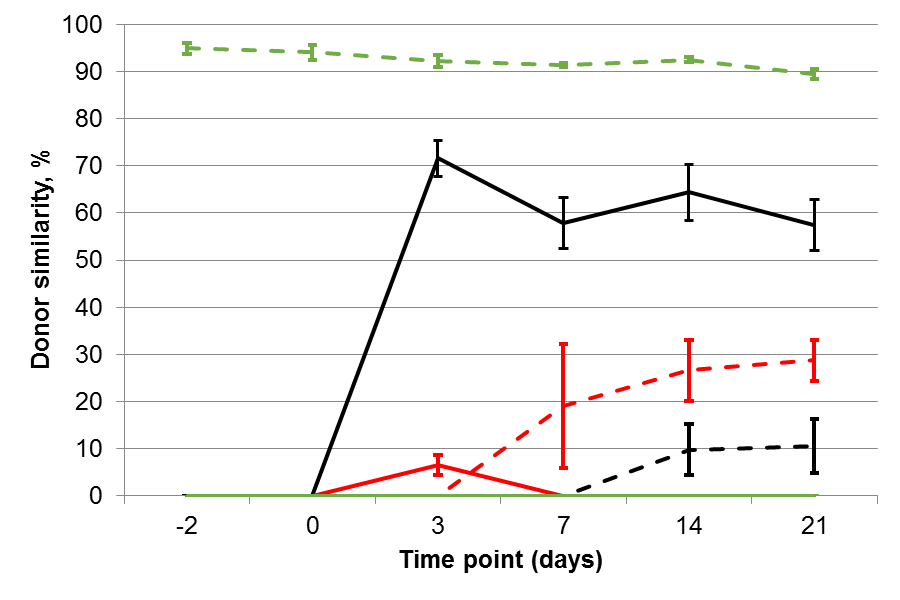


**Figure S7.** Similarity to donor and pre-antibiotic mouse communities among mice treated with three courses of antibiotics. Solid lines reflect similarity to donor 41 and dashed lines reflect similarity to pre-antibiotic mouse communities. Black lines are the experimental group that received both antibiotics and donor gavage, red lines indicate mice that received only antibiotics without donor gavage, and green lines indicate mice that received donor gavage without antibiotic exposure. Error bars reflect standard error.

**
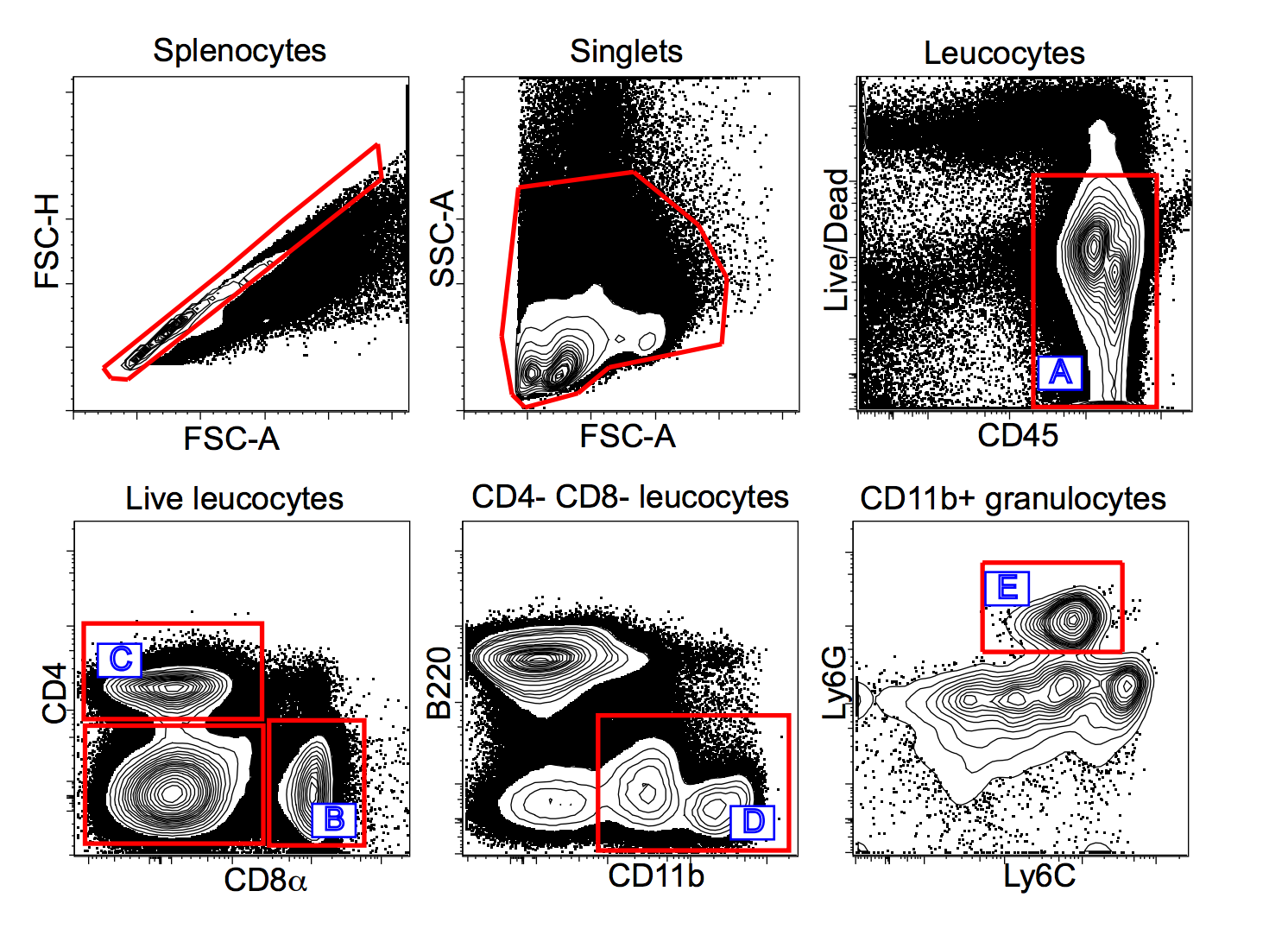
**

**Figure S8**. Flow cytometry gating scheme to identify immune cell populations. Isolated splenocytes were examined using 14-parameter flow cytometry panel. A representative example with indicated populations (on top) is shown. The letter inside the gates correspond to various leucocyte populations enumerated in the next figure. (A) CD45+ leucocytes, (B) CD8a+ T cells, (C) CD4 T cells, (D) CD11b+ granulocytes and (E) neutrophils.


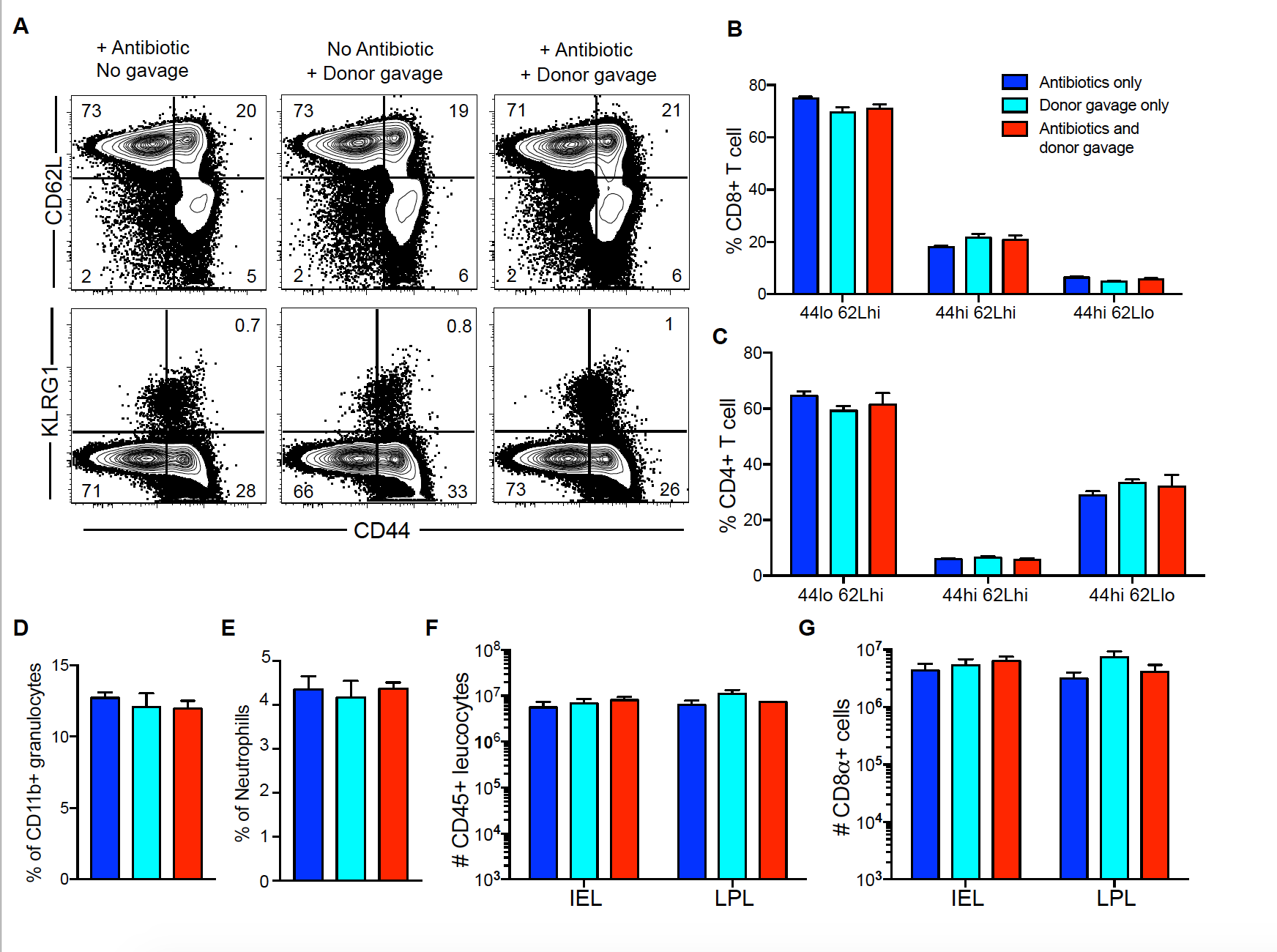


**Figure S9. Immunological parameters in mice are not altered after human microbiota engraftment**. C57Bl/6 mice received oral gavage of human microbiota as described in methods and Figure 4 with or without prior multiple course antibiotic treatment. After verifying successful colonization, T cell differentiation and innate leucocytes composition were assessed. A) Representative flow cytometry plots depicting phenotypes of CD8+ T cell isolated from spleen among the three indicated groups of mice. Bar graphs representing the frequency of major CD8+ T cell subsets (B) and CD4+ T cell subsets (C) in spleen of the different mice groups determined by fluorescence flow cytometry. Bar graph showing the frequency of CD11b+ granulocytes (D) and Ly6g+ neutrophils (E) in peripheral blood of mice. Calculated as a percentage of total CD45+ leucocytes by flow cytometry. Bar graph comparing the number of CD45+ leucocytes (F) and CD8α+ cells (G) in small intestine intra-epithelial compartment (IEL) and lamina propria fraction (LPL). Data are shown for the engraftment experiment done with microbiota from donors 36 and 42 (these recipients were sacrificed on the same day); the data are similar to a separate engraftment experiment with microbiota from donor 41. The bars indicate the mean (6 mice) ± S.E.M. None of the comparisons were significantly different. Kruskal-Wallis one-way analysis of variance.


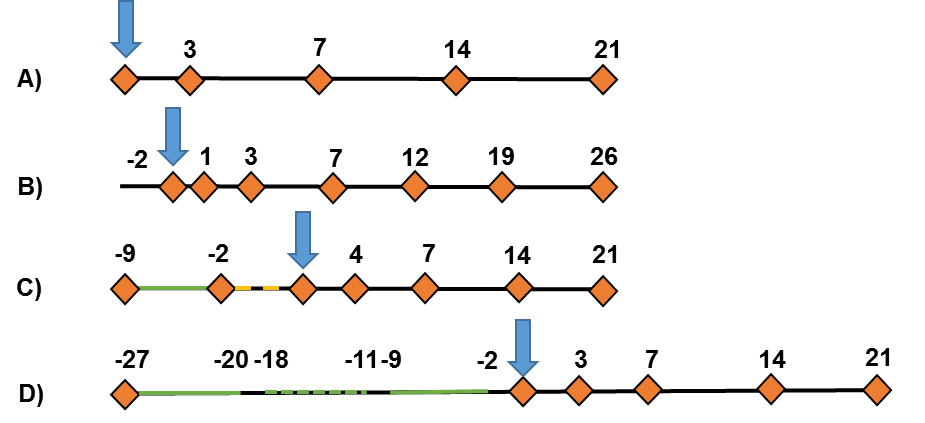


**Figure S10. Timeline of mouse experiments for A) germ-free, B) ASF, C) single-course antibiotic, and D) three-course antibiotic experiments.** Legend: orange triangles – sample collected, blue arrows – gavage performed (T_0_), green bars – antibiotic exposure (dashed lines indicate a different antibiotic cocktail), yellow bars – washout with purgative. Numbers indicate days relative to gavage.

**Table S1.** Distribution of abundant families among germ-free and antibiotic-treated mice that received gavage with the same lot from donor 41.

|  | **Germ-free** | | **3×-antibiotic-treated** | |
| --- | --- | --- | --- | --- |
|  | **T_3_** | **T_21_** | **T_3_** | **T_21_** |
| *Porphyromonadaceae* | 25.27 ± 2.21 | 27.85 ± 8.80 | 53.77 ± 2.03 | 22.32 ± 3.90 |
| *Bacteroidaceae* | 36.64 ± 6.24 | 27.73 ± 9.00 | 10.27 ± 1.44 | 27.67 ± 3.52 |
| *Lachnospiraceae* | 15.44 ± 2.35 | 13.97 ± 2.32 | 15.65 ± 0.11 | 17.73 ± 6.53 |
| *Ruminococcaceae* | 2.48 ± 1.48 | 9.82 ± 1.13 | 2.88 ± 0.03 | 2.24 ± 0.11 |
| *Sutterellaceae* | 3.55 ± 1.26 | 6.39 ± 1.16 | 4.05 ± 0.37 | 4.12 ± 0.84 |
| *Desulfovibrionaceae* | 3.52 ± 0.62 | 2.73 ± 0.10 | 1.82 ± 0.69 | 2.74 ± 0.45 |
| *Erysipelotrichaceae* | 0.67 ± 0.11 | 3.27 ± 0.08 | 1.73 ± 1.80 | 4.85 ± 1.42 |
| *Rikenellaceae* | 0.27 ± 0.12 | 2.14 ± 0.71 | 1.70 ± 0.35 | 2.62 ± 0.46 |
| *Lactobacillaceae* | 0.00 ± 0.00 | 2.53 ± 0.20 | 1.53 ± 1.65 | 4.87 ± 0.64 |
| *Acidaminococcaceae* | 0.14 ± 0.06 | 0.06 ± 0.01 | 3.06 ± 1.86 | 5.96 ± 1.44 |
